# Complete mitochondrial genome of *Ricania shantungensis* Chou & Lu, 1977 (Hemiptera: Ricaniidae)

**DOI:** 10.1080/23802359.2021.1955763

**Published:** 2021-10-20

**Authors:** Hyobin Lee, Keon Hee Lee, Jeong Sun Park, Gwan-Seok Lee, Iksoo Kim, Wonhoon Lee

**Affiliations:** a Department of Plant Medicine, Gyeongsang National University, Jinju, Republic of Korea; b Department of Applied Biology, College of Agriculture and Life Sciences, Chonnam National University, Gwangju, Republic of Korea; c Department of Agro-food Safety and Crop Protection, Crop Protection Division, National Institute of Agricultural Sciences, RDA, Wanju, Republic of Korea; d Institute of Agriculture and Life Science, Gyeongsang National University, Jinju, Republic of Korea

**Keywords:** *Ricania shantungensis*, mitochondrial genome, Ricaniidae, Korea

## Abstract

*Ricania shantungensis* Chou & Lu, 1977 (Hemiptera: Ricaniidae), is an invasive pest that attacks forest as well as agricultural trees. We sequenced the 15,358 bp long complete mitochondrial genome (mitogenome) of this species; it consists of a typical set of genes (13 protein-coding genes, 2 rRNA genes, and 22 tRNA genes) and one major non-coding AT-rich region. The orientation and gene order of the *R. shantungensis* mitogenome are identical to that of the ancestral type found in majority of the insects. Bayesian inference (BI) phylogeny placed the *R. shantungensis* examined in our study, together with *Ricania* spp. in a group with the highest nodal support, forming the family Ricaniidae to which *R. shantungensis* belongs.

*Ricania shantungensis* Chou & Lu, 1977 (Hemiptera: Ricaniidae), is native to China (Chou and Lu 1977) and was detected as an exotic species in South Korea in 2011 (Rahman et al. 2011). Until now, host plants of *R. shantungensis* has been are known to be 138 species belonging to 62 families, including chestnut, peach, persimmon, apple, black locust, Japanese angelica tree, snowbell, pussy willow, paper mulberry, silk tree, tree of heaven, and Japanese cornlian cherry in Korea (Kim et al. 2015).

In previous studies, mitochondrial *cytochrome c oxidase subunit I* (*COI*) were analyzed from specimens collected from Korea; however, these specimens revealed that genetic difference was not associated with geographical distance, suggesting the need for variable markers for population genetics data (Kwon et al. 2017).

## Materials and methods

One wild male adult sample was collected on the tree of heaven (*Ailanthus altissima*) by Hyobin Lee with an insect net in the Haenam-gun, Jeollanam-do, Republic of Korea (34.6724270 N, 126.617252 E) and its DNA was extracted from one of the hind legs. Leftover DNA and the specimen were deposited at the Gyeongsang National University, Jinju, Korea, under the accession number GNUCH08(M5). Using the extracted DNA, four long overlapping fragments (LF1: *COI-ND3*, LF2: *ATP6-ND4*, LF3: *ND5-CytB*, LF4: *ND6-srRNA*, LF5: *IrRNA-COI*) were amplified using four sets of primers designed using data regarding the previously published species of Ricaniidae (*Ricania marginalis*, NC_019597.1; *Ricania speculum*, NC_031369.1). Using the LFs as templates, 36 overlapping short fragments (SF) were amplified using the aforementioned primers.

The location and fragment size of protein coding genes (PCGs), tRNA genes, rRNA genes and AT-rich region were determined based on the published mitochondrial genomic of *R. marginalis* and *R. speculum*. tRNAscan-search server (http://lowelab.ucsc.edu/tRNAscan-SE/) and MITOS web server (http://mitos.bioinf.uni-leipzig.de/index.py) were applied to predict the tRNA genes secondary structure.

**Figure 1.**
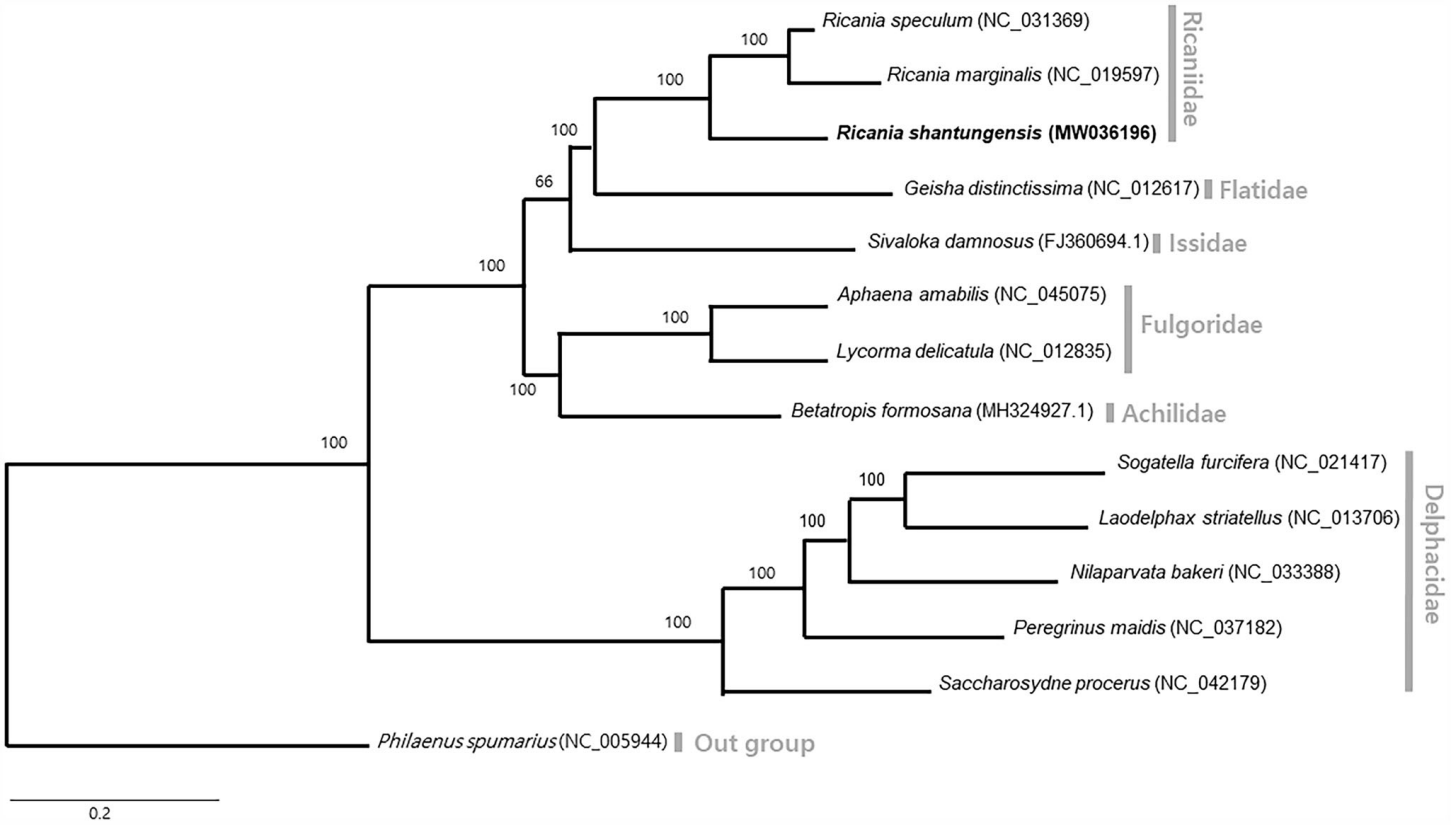
Bayesian inference (1,000,000 generations) phylogenetic tree of 13 Fulgoromorpha mitochondrial genomes. Numbers above branches indicate the posterior probability values of Bayesian inference. Families were displayed with gray bars in the right side of phylogenetic tree.

A phylogenetic analysis was performed using 13 available mitogenomes from Fulgoroidea, including the one obtained in this study (Figure 1). Nucleotide sequences of all PCGs and rRNAs were aligned by using MAFFT (Kazutaka 2005), and 13 PCGs and 2 rRNAs were concatenated in alignment (15,023 bp). Bayesian inference (BI) method was applied using MrBayes version 3.2.6 (Ronquist et al. 2012). A phylogenetic tree was visualized using FigTree version 1.42 (http://tree.bio.ed.ac.uk/software/figtree/).

## Results

The *R. shantungensis* mitogenome was found to be 15,358 bp in length, with typical gene sets − 2 rRNAs, 22 tRNAs, and 13 PCGs – and a major non-coding AT-rich region of 961 bp length (GenBank accession number MW036196). The gene arrangement of *R. shantungensis* was identical to that of the ancestral type found in majority of the insects (Boore 1999).

Phylogenetic analyses using a BI method, using 13 PCGs and 2 rRNAs, placed *R. shantungensis* from Korea, along with *Ricania* spp., into the genus *Ricania*, with the highest nodal support. The family Ricaniidae, to which *R. shantungensis* belongs, forms a cohesive monophyletic group with the highest nodal supports indicated by BI analysis.

## Data Availability

The genome sequence data that support the findings of this study are openly available in GenBank of NCBI at (https://www.ncbi.nlm.nih.gov/) under the accession no. MW036196. The associated BioProject and Bio-Sample numbers are PRJNA700097 and SAMN17823576, respectively.
